# Relationships between Efflux Pumps and Emergence of Heteroresistance: A Comprehensive Study on the Current Findings

**DOI:** 10.1155/2022/3916980

**Published:** 2022-09-19

**Authors:** Mohammad Abavisani, Mansoor Kodori, Fariba Akrami, Ali Radfar, Ali Hashemi

**Affiliations:** ^1^Student Research Committee, Mashhad University of Medical Sciences, Mashhad, Iran; ^2^Noncommunicable Diseases Research Center, Bam University of Medical Sciences, Bam, Iran; ^3^INRS-Centre Armand-Frappier Sante Biotechnologie, Laval, Quebec, Canada; ^4^Department of Internal Medicine, School of Medicine, Bam University of Medical Sciences, Bam, Iran; ^5^Department of Microbiology, School of Medicine, Shahid Beheshti University of Medical Sciences, Tehran, Iran

## Abstract

Heteroresiatnce (HR) is the type of resistance toward one or more antibiotics appearing as a population of the bacterial load consisting of one or more subpopulations with lower antibiotic susceptibility levels than others. Due to the lack of appropriate diagnosis of HR isolates and their importance in resistance emergence to antibiotics, investigating the origins, emergence factors, and HR inhibitors is critical in combating antibiotic resistance. Efflux pumps (EPs) are bacterial systems that own an influential role in acquiring resistance toward anti-bacterial compounds. Studies on EPs revealed that they can affect HR emergence mechanisms and are competent to be introduced as a suitable bacterial target for diagnostic and therapeutic development in combating HR isolates. This review will consider the relations between EPs and the emergence of HR isolates and discuss their importance in confronting this type of antibiotic resistance.

## 1. Introduction

Studies performed about the activity of streptomycin on *Haemophilus influenzae* in 1948 resulted in an observing new phenomenon of antibiotic resistance for the first time [1]. The new occurrence was introduced as heteroresistance (HR). HR is the situation of resistance toward an antibiotic in which a single isolate contains both types of bacterial populations: populations' own susceptibility to antibiotics and populations with resistance toward it [2]. HR is attributed to a heterogeneous population of bacteria, including one or more subpopulations with higher antibiotic resistance levels than others.

This type of resistance appears to own the main difference from the conventional type of antibiotic resistance named homoresistance in which all bacterial loads in a single isolate show resistance toward tested antibiotics. On the other hand, routine diagnostic procedures cannot detect heteroresistant bacterial isolates. Therefore, there is a lack of diagnosis with conventional antibiotic resistance tests, and if operators do not know how to recognize them, they may report them as susceptible isolates toward a specific antibiotic [2]. The importance of HR will be more prominent considering that the heteroresistant isolates can emerge during the acquiring resistance process [3]. They may be an intermediate stage in the emergence of resistant isolates. Thus, emerging and evolving the heteroresistant isolates can impede the combating operation against the worldwide antibiotics resistance crisis.

Identifying the efficient factors in arising the heteroresistant isolates is the first and one of the main ways to constrain them. Efflux pumps (EPs) are the structures among bacterial cells expelling anti-microbial compounds directly into the extracellular space by creating a channel across the cell envelope [4]. These systems cause resistance to broad types of antibiotics and are effective in emerging multidrug-resistant (MDR) and extensively drug-resistant (XDR) isolates of bacteria [5–7]. Considering and investigating the dimensions of EPs leads to the description of these resistance structures as factors influencing heteroresistant emergence.

Consequently, comprehensively studying the relation between EPs and HR occurrence and interactions of efflux systems with mechanisms emerging HR is influential in introducing and developing HR diagnostic methods and can also improve the inhibitors affecting heteroresistant isolates. In the current study, we will discuss the HR and attributed emergence mechanisms, and subsequently, the role of EPs in the appearance of heteroresistant isolates will be reviewed.

## 2. HR Definition and Origins

HR is a phenotype defined as the presence of one or more subpopulations with lower susceptibility to antibiotics compared to the main population [8]. Nicoloff and co-workers considered HR as the presence of subpopulation at frequencies of 1 × 10^−7^ or higher, based on the mutational occurrence rates of <10^−7^ precell per generation [9]. Besides, their resistance level has been considered twofold to eightfold higher than the dominant population's resistance level and minimum inhibitory concentration (MIC) level [2].

**Figure 1:**
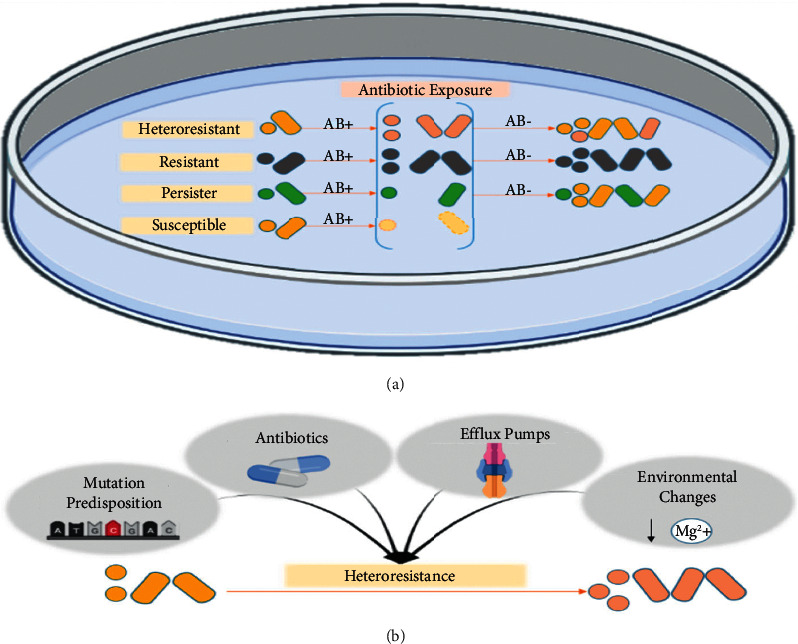
The difference between HR, resistance, persistence, and susceptibility and influential factors in the emergence of HR. (a) In the presence of antibiotic agents, heteroresistant cells can survive and grow in the presence of the antibiotic. Since the HR phenotype is unstable, cells return to the susceptible cell phenotype without antibiotic agents. On the other hand, resistant cells grow in the presence of antibiotics and remain resistant in their absence because genetic changes in resistance are stable, unlike HR. Persistence and HR are both subpopulation-mediate resistance; however, although persister cells can survive antibiotic treatment, they do not grow or grow slowly in the presence of antibiotic agents. In the absence of the antibiotic treatment, persister cells also switch back to the susceptible cell phenotype. (b) Several factors have been identified and hypothesized to be influential in HR emergence: antibiotic exposure, mutation susceptibility in some strains, environmental changes such as decreasing specific ions, and changes in expression of the EPs.

Antibiotic persistence and HR are both subpopulation-mediate resistance [10]. However, the former is the ability of a fully susceptible bacterial subpopulation to survive a high concentration of antibiotics with a temporarily quiescent or shallow growth rate [11]. Persister cells constitute a small portion of the main population that can survive but cannot grow in the presence of antibiotic agents. In the absence of antibiotics, these cells can switch back to susceptible cells [12]. On the other hand, heteroresistant cells can survive and grow under antibiotic pressure. Unlike resistance, the monoclonal heteroresistant phenotype is mainly unstable, and changes are reversed when the stress is eliminated (Figure 1(a)) [12].

HR subpopulations can have different origins and clonality, level of resistance, frequency of resistant subpopulations, and stability in the absence of antibiotics. Subpopulations could be the result of co-infecting clones. Polyclonal HR is linked to either secondary infection by a resistant isolate like what happens in *Helicobacter pylori* and *Mycobacterium tuberculosis* (MTB) HR populations or the emergence of spontaneous very rare resistant mutants, which increase due to antibiotic pressure. Polyclonal HR cannot be found in purified clones; thus, anti-microbial susceptibility tests (AST) would detect a fully susceptible or fully resistant phenotype. Alternatively, monoclonal HR occurs as the individual gets infected by a single clone that differentiates into two distinct high-frequency populations [2]. In contrast to the former, an HR phenotype could be detected in a purified clone [2]. Any cell from the resultant population forms a new population that again displays HR. The level of a new resistance cell line decreases when the antibiotic selective pressure dissolves because the phenotypic changes are unstable and/or costly. Thus, the transient increase in MIC values is also unstable and reversible [12].

Monoclonal HR can have a phenotypic or genetic basis. The most frequently unstable genetic changes are resistance gene amplification to cause genetic-based monoclonal HR. However, these changes are volatile and costly, easily lost in the absence of selective antibiotic pressure [12]. A typical example of phenotypic monoclonal HR against colistin can be seen in clinical isolates of *Enterobacter cloacae* following the modification of the outer membrane lipopolysaccharide (LPS) following activation of the PhoPQ two-component system (TCs) [2]. The activation of *pmrAB* and *phoPQ* TCs also reported in HR against *Escherichia coli, Pseudomonas aeruginosa*, and *Klebsiella pneumoniae*. Biofilm formation in *K. pneumoniae* and putrescine/YceI communication in *Burkholderia cenocepacia* are several mechanisms that result in colistin HR [13]. Resistance mutations with low or no fitness costs are more likely to be stable without selective pressure. In several studies, the resistance mechanism in stable HR involved efflux and/or influx of antibiotics. Increased expression of EPs and decreased membrane permeability have been reported in stable resistance *P. aeruginosa* subpopulations against carbapenems [14, 15]. Several genes and mechanisms are involved in EPs in different species (see later).

### 2.1. Clinical Importance and Complications in Treatment

HR can lead to treatment failure, especially in MDR and XDR species, defined as persistent infection or bacteremia and/or continuing the infection signs, by increasing the frequency during antibiotic exposure [16, 17]. Persistent bacteremia can lead to high-inoculum infections such as deep abscesses, osteomyelitis, and infective endocarditis in heteroresistant vancomycin-intermediate *Staphylococcus aureus* (hVISA) isolates [16]. Also, it has been shown that carbapenem-heteroresistant *K. pneumoniae* strains producing extended-spectrum *β*-lactamases (ESBL)/AmpC *β*-lactamases inhibit imipenem (IMP therapy in experimentally infected mice [18].

HR may facilitate the evolution of permanent resistance through the accumulation of mutations. The initial increase in MIC could augment the adaptive benefit of individual resistance mutations [12]. The emergence and extinction of different subpopulations triggered by therapy changes and suboptimal treatment regimens can lead to the fixation of resistance mutations. Sonnenkalb et al. reported a patient who had received 27-year treatment for MTB ending with a nearly untreatable XDR-MTB due to fixation of mutations in the long term [19]. Identifying HR infections prior to the treatment and following antibiotic concentration modification may reduce the possible failure in treatment and the occurrence and extension of complete drug-resistant isolate [20].

Diagnosis of the heteroresistant phenotype is crucial to choosing the best treatment regimens [2]. It is also essential to define the HR level that affects clinical outcomes. Chen et al. showed that resistant subpopulations that are lower than 1% of the total bacterial colonization in MTB had a minimal effect on therapeutic outcomes and the immune system can control <1% of the total bacterial load [21]. It was shown that most heteroresistant strains could be heteroresistant to more than one antibiotic [3, 8, 11]. HR against amikacin and meropenem (MEM) in the *K. pneumoniae* strain has been reported in China [17]. These infections can be eliminated by using a combination antibiotic therapy that has been proven to be effective *in vivo* and *in vitro* [12]. Some studies have concluded that these combination antibiotic regimens are ineffective and do not have superiority over monotherapy [22]. However, *in vivo* studies have shown the efficacy of combination therapy when antibiotic monotherapy fails. Amikacin combined with MEM combination therapy against *K. pneumoniae* strain SWMUF35 showed co-HR toward both antibiotics [17]. Another example is polymyxin B (PMB) and tigecycline (TGC) combination against carbapenem-resistant *K. pneumoniae* (CRKB) HR to one or both of the antibiotics that are proposed to be a treatment strategy for the infection [22].

Since overexpression of different EPs genes has been found to associate with HR in several species, using efflux pump inhibitors (EPIs) can have a synergist effect with other agents. Examples of this strategy were shown in combination therapy of *Acinetobacter baumannii* HR strains with efflux inhibitors and colistin and ethidium bromide and administration of carbonyl cyanide m-chlorophenylhydrazone (CCCP) as an EPs inhibitor to reverse HR against several Gram-negative bacteria (GNB) including *E. coli* [13, 14, 23].

### 2.2. HR Diagnosis

It is hard to detect HR isolates because of the phenotypic and genetic instability of heteroresistant subpopulations(s) [24]. Routine antibiotic susceptibility methods may misinterpret the HR phenotypes [22]. Most of the available methods require bacterial growth as a readout, which takes time, and treatment planning cannot often be postponed [12]. The most reliable test that is considered the gold standard for detecting HR is population analysis profiling (PAP); however, the methods for conducting and interpreting the result of this test have not been standardized yet [25]. It involves counting colony-forming units (CFU) in media with an increasing concentration of the antibiotic agent compared to growth in the absence of the antibiotic [8]. PAP is an expensive, labor-intensive, and time-consuming test, limiting its use to confirm specific clinical cases [2].

The population analysis profile-area under the curve (PAP-AUC) method is cheaper and faster than the PAP test, and it is the current gold standard for detecting hVISA. However, it still cannot be implanted in routine clinical settings. Other alternative procedures, the disc diffusion assay and Epsilometer test (E-tests) have serious limitations of not being quantitative and showing high frequencies of false positive or false negative samples [2, 16, 24]. Culture-independent and biochemical methods, such as VITEK2, and PCR-based methods, such as the Xpert MTB/RIF, have been developed [2, 12]. However, the lack of genetic markers and low sensitivity make these methods insufficient to detect HR [24].

Whole-genome sequencing (WGS) is proposed as an accurate, promising, and affordable method compared to the traditional phenotype tests to detect resistant subpopulations in clinical isolates, with limitations of detection of the frequency of the subpopulations less than <1% [2, 21]. Although studies on MTB and *S. aureus* have shown promising results, this method has failed to detect anti-microbial resistance from HR subpopulations in *Salmonella enterica*. Authors suggested that widespread use of WGS may underestimate the actual resistance rate [26–28]. Monitoring metabolic activity measured by heavy water (D2O) uptake in *E. coli* and *Enterococcus faecalis* is an extended Raman-based susceptibility test developed by Bauer and co-workers [29].

Droplet digital PCR (ddPCR) detects genes or point mutations involved in resistance in heteroresistant subpopulations, and it was used to detect resistant *H. pylori* with promising results [30]. Most recently, Pitruzzello et al. proposed an HR (monoclonal and polyclonal) detection and MIC quantification method by using bacterial motility at single-cell resolution in *E. coli* and *S. typhimurium* [31]. However, new, fast, and feasible diagnostics with high reproducibility and low cost are needed with favorable sensitivity to detect the low amount of resistant cells [10].

### 2.3. The Effective Factors in the Emergence of HR

Different factors can induce the probability of the emergence of HR (Figure 1(b)). Antibiotic exposure has been known to be a significant factor in the emergence of HR. Studies on *A. baumannii*-related carbapenems exposure observed HR emergence [32]. Similarly, *S. aureus* exposure to vancomycin may induce HR against daptomycin [33]. *In vivo* and *in vitro* studies have shown that prior colistin exposure results in a higher frequency of resistant subpopulations, but HR was detected in isolates without prior exposure. Many studies suggested that HR may be a strain-specific characteristic, and some strains are more prone to develop chromosomal mutations that lead to HR [25]. Also, the frequency of resistance mutations, the fitness cost of such mutations, and the frequency of compensatory mutations that do not affect the phenotype highly impact the *in vivo* emergence of HR [2]. Environmental changes may also induce HR. For example, *E. cloacae* cells survive colistin by modifying the LPS component of their OM, and *phoPQ* TCs regulate this process. This system is activated by decreasing Mg^2+^ environmental ions [12]. Overexpression of several EPs such as *oqxAB* and *macAB* and their transcriptional regulators were also suggested to be associated with HR in strains such as *K. pneumoniae, S. enterica, and E. cloacae* [34–36].

## 3. Bacterial EPs and Antibiotic Resistance

In the periplasm or cytoplasm bacterial cell, EPs identify harmful substrates that have pierced the defensive cell wall and expel them before reaching their desired sites or considerable concentration [37]. Bacterial EPs proteins conduct excretory functions to cope with various hazardous compounds and harmful metabolic waste products regardless of internal or external origin and transfer them from within cells into the external environment [38]. There are two main groups of EPs based on their energy source: ATP hydrolyzers and proton motive force (PMF) utilizers. PMF class contains MF (major facilitator), MATE (multidrug and toxic efflux), RND (resistance-nodulation-division), PACE (proteobacterial anti-microbial compound efflux) family, and SMR (small multidrug resistance). In contrast, the ATP-binding cassette (ABC) superfamily exploits ATP hydrolysis for the transporter's conformational modifications and influx or efflux of the diverse substrate (metabolites, vitamins, ions, peptides, lipids, amino acids, and drugs) [39]. Additionally, types of these transporters are distinguished by nucleotide-binding domains (NBDs) and transmembrane domain (TMD) and are commonly inhibited by arsenate, which reduces cellular ATP. However, MFS transporters are relatively insensitive to arsenate [37]. Each of the bacterial genomes (susceptible or resistant strains) examined comprises multiple EPs encoded on plasmids and other transmissible elements or chromosomes. It is assessed that between 5% and 10% of bacterial genes are implicated in transport, with a considerable fraction encoding EPs. Those have been maintained during evolution paths, especially the MFS, MATE, and RND families [40]. In contrast to the ABC transporters, members of the RND family derive their energy from the PMF formed during cellular metabolism [41]. Studies revealed that increased expression of EPs may be associated with elevated MIC or resistance to some antibiotics like over-expression of *acrAB* in *E. coli*. EPs can be overexpressed through mutations in local repressor genes or through activation of a regulon controlled by a global transcriptional regulator such as MarA or SoxS in *E. coli*. For instance, 98 *E. coli* isolates were discovered to have efflux pump-mediated carbapenem nonsusceptibility in a study conducted by Chetri et al. in 2019. Ertapenem resistance and *acrA* overexpression were shown to be strongly correlated, which has not been previously reported. Additionally, it was found that *E. coli acrB* expression was elevated by imipenem stress [42], and in another survey, every single isolate of *E. coli* was drug-resistant (MDR). Together, 48 (96%) of the isolates had resistance to more than three antibiotics, and 2 (4%) of them had resistance to three drugs. The *acrAB*-*tolC* EPs in *E. coli* and these genes were detected in (98%) of isolates by utilizing the PCR technique [43]. Cross-resistance was also observed in certain therapeutically significant antibiotics, such as the *P. aeruginosa mexAB* overexpression system, resulting in decreased susceptibility to other antibiotics. Additionally, overexpression of multidrug-resistant EPs increases bacteria' chances to survive and additional alterations in genes encoding antibiotic target sites [40]. RND family systems are significant EPs in GNB and are involved in antimicrobial resistance (AMR) to a variety of types of antibiotics, including fluoroquinolones, cephalosporins, tetracyclines, aminoglycosides, penicillins, and macrolides. RND systems are typically tripartite (three components) and are structured differently and span the OM, inner membrane (IM), and periplasm. *MexAB*-*oprM* from *P. aeruginosa* and *acrAB*-*tolC* from *E. coli* are the two most well-characterized RND types. Homologs of *acrAB*-*tolC* have been discovered in members of the *Enterobacteriaceae* and other GNB, and its overexpression provides resistance to bile and a variety of substrates, including antibiotics and biocides, dyes, detergents, and fatty acids, as well as solvents [42]. Several investigations have found that HR subpopulations with stable resistance mechanisms involve antibiotic efflux and/or influx. In the following, we bring EPs mechanisms involved will be discussed.

### 3.1. Heteroresistant Bacteria

#### 3.1.1. P. aeruginosa 

*P. aeruginosa* is a Gram-negative, nonfermenting, opportunistic organism responsible for various infectious diseases [43]. This bacterium is highly resistant to multiple antibiotics classes and is a leading cause of nosocomial and hospital-acquired diseases with a high mortality rate. This intrinsic or developed resistance has twisted anti-pseudomonal treatment [44]. Besides resistance and persister cells, HR is a transitional stage between susceptible and whole resistance cells, and several studies have indicated its association with patients' treatment and clinical consequences [45–47]. This particular resistance type can interfere with accurate clinical detection and may result in clinical anti-infection failure [9]. Up to this point, several potential mechanisms for HR-P. aeruginosa have been described, including TCSs, AmpC *β*-lactamase, EP overexpression, certain operons (OprD), biofilm formation, etc. [15, 45, 48, 49]. In this section, we described the function of EPs in the emergence of HR-*P. aeruginosa.*

Mei et al. determined HR to IMP in *P. aeruginosa* strains obtained from hospitalized patients in China. Their result revealed that 18.87% had HR to IMP and remained resistant after five generations of subculture. It indicated that higher mutation frequency associated with IMP-HR varied between 6×^−7^ and 4.5×^−9^, which needs more consideration in clinical practice. Further analysis specified that HR strains have higher *MexAB* expression levels than IMP susceptible strains and none of which encode Metallo-*β*-lactamase (MBLs). However, this significant difference was not found in the expression level of *MexCD*. It has been suggested that the HR may be unstable in some *P. aeruginosa* strains (Table 1) [9]. Another report from China declared IMP-HR rate was 35.1% among clinical *P. aeruginosa* strains. It is also worth noting that 3 two BP deletion at 1116–1118 and 1147–1149 of *oprD* has an essential function in developing HR and resistance strains and should be carefully factored in when evaluating drug-resistant. *OprD* premature stop translation was identified in all resistance isolates but observed less frequently in HR isolates than resistant isolates. The MICs of other antibiotics revealed a slight change in IMP-HR isolates. As a result, IMP combined with other antibiotics may be more beneficial for treating IMP-HR-*P. aeruginosa* isolates than IMP alone. As previously noted, *oprD* expression was lower than the parental strain, and a relationship between *mexE* and *mexY* was seen between two IMP-HR and MEM nonresistant strain groups. On the other hand, IMP-resistant and HR-*P. aeruginosa* isolates did not exhibit significantly different resistance mechanisms. It may be due to varied resistance patterns and other external triggers like IMP or antibiotic stress [55]. Similarly, Ikonomidis et al. analyzed four genetically unrelated heterogeneous carbapenems of *P. aeruginosa* clinical isolates and observed that all these isolates have fourfold higher MICs for IMP and MEM than native cells. They reported that *mexB* and *mexY* gene expression had increased dramatically, while *oprD* transcription had decreased, and the *mexE* gene expression had remained constant. PAP test of carbapenem apparently susceptible isolates showed that HR subgroups could grow at a frequency of 6.9 × 10^−5^ to 1.1 × 10^−7^ in higher MICs than individual native isolates, which may go undetected under traditional agar dilution MIC testing. As a result, resistant *P. aeruginosa* subpopulations may be selected, leading to infections and treatment failure [56]. Other studies on *P. aeruginosa* from invasive infections over five years demonstrated a significant carbapenem heteroresistance (CHR) (84.9%), steadily increasing yearly. It may respond to numerous carbapenem usage and selection pressure the HR in this study. Of the EPs, *mexB* and *mexE* have overexpression, and a significant correlation was observed with IPM-HR. In addition, prior carbapenem exposure has been reported as the most common independent risk factor for developing IPM-HR and MEM-HR. Furthermore, biofilm formation was suggested to contribute to the CHR emergence in *P. aeruginosa* strains [47]. A comparison of *P. aeruginosa* strain PA7171 isolated from urinary tract infection (UTI) with ATCC 27853 revealed that this HR strain could grow with a frequency of 10^−7^ in a high dose of piperacillin/tazobactam (128 mg/L); nevertheless, both strains exhibit a similar bactericidal curve [50]. According to limited EPs investigations, the HR prevalence of antibiotics in *P. aeruginosa* isolates differs, which may be linked to higher expression of the *mex* genes.

#### 3.1.2. A. baumannii

*A. baumannii* is a common nosocomial pathogen that causes life-threatening infectious diseases in hospitals and communities. It is an associate of the ESKAPE group pathogens and has inherent resistance to multiple antibiotics and has the ability to develop new resistance determinants readily [57]. This remarkable resistance spells trouble in the treatment of *A. baumannii* infections. Over and above that, HR-*A. baumannii* was described across several investigations from various regions and may be selected and become predominant during therapy and complicating the treatment of MDR strains [7, 58, 59]. TGC-HR-*A. baumannii* was examined in a collection of clinical isolates from South Korea. It was reported that the efficacy of TGC would be decreased in the existence of HR strains. The time-kill assay revealed all HR was not killed at twice MIC, and some of these HR strains can regrow during antibiotics therapy. All HR subpopulation has the insertion of *ISAba1* in the *adeS* gene at different locations. This interruption leads to a truncated soluble *AdeS* protein generation and the *adeB* and *adeS* upregulation and emergence of HR subpopulation to TGC. This resistance phenotype was not stable in an antibiotic-free medium, and the MIC of strains was reduced to different degrees. Moreover, in this medium, expression levels of *adeB* and *adeS* were downregulated and highlighted the role of the *adeABC* EP in the emergence of HR *A. baumannii* to the TGC [7]. Colistin HR analysis in strain variants indicated *adeB*, *adeJ*, *adeG*, *craA*, *amvA*, *abeS*, and *abeM* EP genes were overexpressed in response to colistin exposure. Antibiotic therapy combined with efflux inhibitors was also found to be effective in resensitizing *A. baumannii* to colistin and preventing drug resistance [60]. Another study found that the prevalence of colistin HR is substantially lower (1.84%) than previously reported and that the HR subpopulation has weaker biofilm development potential than the ATCC19606 strain. The HR subpopulation analysis revealed that *adeI* and *adeB* were upregulated in some isolates. The addition of an EPs inhibitor (CCCP) significantly reduced the MIC of colistin by more than fourfold, implying a function for EPs in colistin HR [61].

#### 3.1.3. *H. influenzae*

*H. influenzae* is an opportunistic pathogen that infects humans and causes both acute infections and chronic illnesses [14]. In 1947, Alexander and Leidy observed the first HR in this organism with modified streptomycin susceptibility [52]. Few studies described HR *H. influenzae* clinical isolates; however, due to the method's poor performance, the precise prevalence of these characteristics may be underestimated. It is uncommon to identify isolates with IMP MIC values greater than the susceptible breakpoint, and this scarcity may be addressed by the heterogeneous expression of resistance and the lack of studies that have examined this issue. Due to the lack of a well-established procedure, this common HR subpopulation is ignored during regular broth microdilution [62]. In *H. influenzae*, sequencing analysis of the *ftsI* genes encoding PBP3 showed different mutation types of correspondence with HR and IMP susceptible isolates. Additional analysis of the HR to IMP isolates demonstrated a partial deletion of *acrR*, which resulted in the loss of regulation of the *acrAB*-*tolC* EP and may have been involved in the development of IMP-HR *H. influenzae* [62, 63].

#### 3.1.4. *K. pneumoniae*

*K. pneumoniae* is an opportunistic pathogen that can cause pneumonia, wound infection, UTIs, and other life-threatening conditions in the hospital and community environments [64]. MDR-*K. pneumoniae* is rapidly evolving by acquiring resistance factors, and the World Health Organization (WHO) announced it as a serious matter of concern in 2017 [53]. In clinical isolates of *K. pneumoniae*, the HR subpopulation was reported in response to antibiotics such as colistin, aminoglycosides, tetracycline, and others [65]. This phenotype has the potential to progress to complete resistance and produce an outward appearance of resistance. Several pathways have been discovered in the evolution of HR *K. pneumoniae*, and the EPs function has been outlined [65, 66].

The involvement of EPs in *K. pneumonia* HR has been studied in a few studies. Tian et al. evaluated the TGC and PMB HR subpopulations, as well as the combined HR to both, in CRKP clinical strains from China. PAP analysis revealed that 66.2%, 100%, and 48.4% of susceptible strains were HR to TGC, PMB, and TGC and PMB concurrently. After 10 hours of incubation, the time-kill assay revealed rapid regrowth, and it was noted that PMB or TGC monotherapy was unable to establish a bactericidal effect over prolonged treatment. However, for all strains, combined therapy with PMB and TGC may result in an early bactericidal effect, even at lower doses. In comparison to their native strains, HR TGC strains demonstrated increased expression of TCs (*phoP* and *pmrA* genes) and the *acrAB*-*tolC* EP (*acrB* gene). The cultivation of HR strains in an antibiotic-free medium indicates that this phenotype is stable, indicating that it may represent an intermediate resistance condition that contributes to treatment failure [67]. In a survey of clinical isolates from China, HR to eravacycline was found in 5.08% of the isolates. When compared to the reference strain *K. pneumoniae* ATCC 13883, these isolates have *oqxAB* and *macAB* overexpression. Furthermore, their findings imply that the *macAB*-*tolC* multidrug EP in *K. pneumoniae* may be involved in eravacycline resistance and HR [34].

#### 3.1.5. *S. enterica*

*S. enterica* serovar typhimurium is an influential food-borne bacterium that can cause a variety of diseases in humans, and the spread of MDR strains, particularly ESBL-producing and fluoroquinolone-resistant strains, is a significant concern worldwide.

The HR research on TGC demonstrates that 14028 strain transformed with the *oqxAB*-bearing pHXY0908 plasmid (IncHI2 type) exhibits a subpopulation with high-level resistance to TGC. In MIC lower breakpoint, this subpopulation is identified at a frequency of 10^−5^ to 10^−8^ compared to 10^−7^ survival in the selection process. The emergence of HR strains with reduced susceptibility was substantially higher than parental (14028 strain) or curing cataing plasmid strains (14028/Δp52 strain). TGC was also found to be less accumulated in HR strains, and overexpression of the *acrAB* and *oqxAB* EPs was detected, which is linked to HR to TGC in the *S. enterica*. Furthermore, mutations in the *ramR* gene cause *ramA* (global regulators that activate *acrAB*) and *acrAB* genes to be overexpressed. Moreover, these HR bacteria had a 4–8 increase in ciprofloxacin MIC, demonstrating a mechanism leading to antibiotic class cross-HR [52].

#### 3.1.6. Enterobacter ssp

*Enterobacter* spp. are opportunistic nosocomial pathogens and create various infections in humans, especially in ICUs patients. Colistin resistance has recently emerged in carbapenem-resistant *Enterobacter* ssp. from multiple countries. However, the exact processes underlying colistin resistance are unknown. Comparative genomic analysis of *Enterobacter asburiae* and *E. cloacae* strains was reportedly conducted to identify a putative colistin HR mechanism. The previously described colistin resistance determinants, *phoP*, *phoQ*, *phoPQ*, *pmrA*, *pmrB*, *pmrAB*, *arnE*, *arnF*, and *arnBCADTEF* mutations, were not identified in this analysis. The Tn5 mutagenesis library demonstrates that the *tolC* gene was inactivated, which resulted in the loss of regular operation of the *acrAB-tolC* EP and a modification in the antibiotic susceptibility profile of the examined *Enterobacter* spp. Additionally, adding PA*β*N to the HR strains culture dramatically reduces the colistin MIC, implying a role for the *acrAB-tolC* EP in colistin HR *Enterobacter* strains. In comparison to the susceptible strain, overexpression of the *acrAB-tolC* cassette genes has been seen in the colistin HR strain. Furthermore, there was no alteration in the transcription of the *mgrB*, *pmr,* and *arnA* genes, ruling out the prospect of lipid A pathway synthesis genes being involved in HR colistin [68].

### 3.2. HR Emergence Mechanisms

According to the latest reports, HR is quite widespread in various bacterial species and antibiotic classes. The HR emerges into types that are polyclonal and monoclonal HR populations. Polyclonal HR emergence under selective antibiotic pressure means that bacteria increase cell numbers to generate mixed infection for escaping from this condition. There are two reasons for acquiring these features among bacteria: (1) presence of susceptible and resistant strains as mixed populations in the infection and (2) emergence of rare spontaneous resistant mutants during anti-microbial treatment. Lung as an infected organ by *H. pylori* and MTB is a prominent example of polyclonal HR phenotype diagnosed through AST [15]. On the other hand, pure clones are responsible for monoclonal HR because of physiological or genetic heterogeneity. There is currently no experimental evidence to suggest the presence of a nongenetic process that causes HR to occur. In a range of bacterial species and antibiotic classes, nongenetic mechanisms have been linked to creating so-called persister cells [51]. Nongenetic means can form these kinds of cells; then the persistent subpopulation does not grow in the presence of drugs but can survive. The primary reason for unstable HR, which contains a mixed population of resistant and susceptible cells, is the reversibility of the resistant phenotype in the absence of antibiotics. Mutations that carry a high fitness cost, including insertions, deletions, and single nucleotide polymorphisms (SNP), can cause unstable HR. This results in a mixed susceptibility population with improved fitness due to compensatory mutations and a rare cell subpopulation with resistance mutations and reduced fitness. Trimethoprim-sulfamethoxazole, carbapenems, and aminoglycosides have all been found to have mutated HR. In the heteroresistant subpopulations of *S. enterica*, *K. pneumoniae*, and *E. coli*, mutations in the genes that encode oxidases and cytochromes in electron transport were found. Recent research has revealed that the typical mechanism for establishing HR in GNB is the unstable amplification of resistant genes, which is lost when cells are cultured in the absence of an antibiotic. [69, 70]. Some mutations that had a minor effect on mutant fitness are kept during culture in a free-antibiotic medium and could result in stable HR subpopulations [46]. Moreover, some studies revealed that increased EPs expression is related to stable HR phenotype such as *mexAB* expression in the HR to IMP in *P. aeruginosa* isolates [9].

### 3.3. Treatment Strategies

Using the time-kill test, researchers demonstrated the efficiency of medication combinations in delaying or suppressing the regeneration of heteroresistant subpopulations. Several screening investigations have found that phenotypic HR is widespread in clinical isolates, while the role of phenotypic HR in treatment efficiency decrease is unknown [15]. Another way is using EPIs in a combination of the drug. For a chemical to be classified as EPIs, it must satisfy the following requirements: (1) it must be able to enhance effluxed antibiotic activity in cultures that express the working pumps, (2) it must not augment anti-microbial activity in strains that do not express the EPs, and (3) it must interact with *AcrA* or *AcrB* [71]. For example, the *norA* gene encodes a 42 kDa protein in the bacterial cell membrane, making it one of the most investigated pumps in *S. aureus. NorA* EP overproduction may be demonstrated in two ways: either by mutations in the genes that code for *norA* or through inducible *norA* expression via regulatory genes. Overexpression of the *norA* gene causes organisms to become more resistant to *norA* substrates, resulting in a variety of antibiotic resistance patterns, including resistance to fluoroquinolones [2, 73]. Nilotinib, a tyrosine kinase inhibitor, was shown to have significant EP inhibitory efficacy, with a fractional inhibitory concentration index of 0.1875 (suggesting a synergistic interaction) and a low MEC of 0.195 M [73]. Today, the RND transporter inhibitor ethyl 4-bromopyrrole-2-carboxylate (RP1) is identified from a library of 4000 microbial exudates. In strains overexpressing archetypal RND transporters, RP1 reduces the minimum inhibitory concentration of antibiotics, working in synergy with them (*AcrAB*-*tolC* and *MexAB*-*oprM*). Hoechst 33342 is likewise better accumulated, and its efflux is inhibited (a hallmark of EPI functionality). Combinations of antibiotics and RP1 lessen the concentration of antibiotics needed to prevent mutations while extending the effects of post-antibiotic therapy [74]. Furthermore, in several therapeutically relevant bacterial species, the capacity of some EPIs to elicit significant reductions in biofilm production *in vitro* has been documented [75]. EPIs should be evaluated for future development because of their direct influence on biofilm production and their indirect potential to potentiate the efficacy of antibiotics.

## 4. Conclusions

Since HR appears to be prevalent in clinical bacterial isolates and may be associated with decreased treatment efficacy and a worse clinical outcome during anti-microbial therapy. It is essential to have a specified operational definition and approach for determining clinical relevance. These isolates may also be heteroresistant to various antibiotics, with a cross-resistant subpopulation. Several mechanisms contributed to the emergence of HR strains in clinically relevant bacteria. The findings gathered in this review indicate that enhanced EPs expression can play a significant role in bacterial resistance to antibiotics at different levels. This occurrence could be caused by a mutation in either the genes regulator in the EPs or the master regulator that governs EPs transcription. Thus, it is essential to comprehend the evolution of the HR phenotype and its impact on treatment failure and clinical outcomes.

## Figures and Tables

**Table 1:** HR prevalence among apparently susceptible bacteria strains and observed correlation with overexpression level EPs.

Bacteria	*N*	Country/isolation source	Antibiotic/heteroresistant rate (%^*∗*^)	Efflux pump	Ref
*P. aeruginosa*	131	China/clinical isolates	IMP/35.1	Increased expression of the *mexE* and *mexY* compared to their respective native ones.	[50]
	106	China/hospitalized patients	IMP/18.87	Higher *mexAB* expression level compared to IMP-susceptible strains.	[15]
	4	Greece/clinical isolates	IMP and MEM/-	Fourfold higher MIC compared to native cells for IMP and MEM	[51]
				Increased transcription levels of the *mexB* and *mexY* genes.	
	451	China/sterile body fluids	Carbapenems/84.9	Identical PFGE profiles HR isolates with native cells	[48]
				HR isolates grew in higher MICs.	
				All HR isolates are negative for MBL production.	
				Upregulation in all HR, including *mexB*, *mexC*, *mexE*, and *mexX* genes.	
				Reduced expression of the *oprD* gene in HR isolates	
				Higher biofilm formation HR isolates	

*A. baumannii*	260	South Korea/clinical isolates	TGC/52	HR isolates have eightfold-higher TGC MICs than the original isolates.	[7]
				In HR isolates *adeABC* efflux pumps are upregulated as a result of *ISAba1* insertion into *adeS* genes.	
	3	Portugal/MDR clinical isolates	Colistin/-	HR isolates have upregulation of *adeB*, *adeJ*, *adeG*, *craA*, *amvA*, *abeS*, and *abeM* Eps genes.	[14]
	576	China/clinical isolates	Colistin/1.84	Colistin HR had weaker biofilm formation capacity than the ATCC19606 strain.	[52]
				Colistin HR has upregulation of *adeI* and *adeB* genes compared to ATCC19606 strain.	

*K. pneumoniae*	74	China/carbapenem-resistant	TGC/66.2	HR isolates have upregulation of *acrB* in the TGC-resistant subpopulations.	[22]
	90		PMB/100		
	56		Both TGC and PMB/48.4		
	393	China/clinical isolates	Eravacycline/5.08	Overexpression of *oqxAB* and *macAB* efflux pumps and the transcriptional regulator *ramA*.	[34]
				Overexpress *acrA*, *acrB*, and *tolC* genes.	
				Mutations in the *acrR* and *ramR* in some HR isolates.	
				*oqxAB* and *macB* mutations in some isolates.	

*H. influenzae*	59	Switzerland/clinical isolates	IMP/77.9	Mutation in the *ftsI* gene (encodes PBP3).	[53]
				Mutation in the OmpP2.	
				Partial deletion of *acrR*.	

*Enterobacter* spp.	—	Laos and Nigeria/-	Colistin/-	Inactivation of *tolC* in the *acrAB-tolC* pump change HR to susceptible strains.	[54]
				In HR strains, overexpression of *acrAB*, *tolC*, and *soxSR* genes was observed.	
				In addition, PA*β*N significantly decreases the MIC of colistin on the HR strain.	

*Note. N*: number, Ref: reference, IMP: imipenem, MEM: meropenem, TGC: tigecycline, and PMB: polymyxin B.

## Data Availability

No data were used to support this study.

## Abbreviations

ABC: ATP-Binding Cassette
AMR: Antimicrobial resistance
AST: Antimicrobial susceptibility test
CCCP: Carbonylcyanide m-chlorophenylhydrazone
CFU: Colony-forming units
CHR: *Carbapenem heteroresiatnce*
CRKB: Carbapenem-resistant K. pneumonia
ddPCR: Droplet digital PCR
EPIs: Efflux pump inhibitors
EPs: Efflux pumps
ESBL: Extended-spectrum β-lactamases
E-test: Epsilometer test
GNB: Gram-negative bacteria
HR: Heteroresiatnce
hVISA: Heteroresistant vancomycin-intermediate Staphylococcus aureus
IM: Inner membrane
IMP: Imipenem
LPS: lipopolysaccharide
MATE: Multidrug and toxic efflux
MBL: Metallo-β-lactamase
MDR: Multidrug-resistant
MEM: Meropenem
MF: Major facilitator
MIC: Minimum inhibitory concentration
MTB: Mycobacterium tuberculosis
NA: Not applicable
NBDs: Nucleotide-binding domains
OM: Outer membranes
PACE: Proteobacterial antimicrobial compound efflux
PAP: Population analysis profiling
PAP-AUC: Population analysis profile-area under the curve
PMB: Polymyxin B
PMF: Proton motive force
RND: Resistance-nodulation-division
SMR: Small multidrug resistance
SNP: Single nucleotide polymorphisms
TCs: Two-component system
TGC: Tigecycline
TMD: Transmembrane domain
UTI: Urinary tract infection
WGS: Whole-genome sequencing
WHO: World Health Organization
XDR: extensively drug-resistant
